# Cysteine cathepsins are altered by flow within an engineered *in vitro* microvascular niche

**DOI:** 10.1063/5.0023342

**Published:** 2020-11-04

**Authors:** Simone A. Douglas, Kristina Haase, Roger D. Kamm, Manu O. Platt

**Affiliations:** 1 Wallace H. Coulter Department of Biomedical Engineering at Georgia Institute of Technology and Emory University, Atlanta, Georgia 30332, USA; 2 Department of Mechanical Engineering and Department of Biological Engineering, Massachusetts Institute of Technology, Cambridge, Massachusetts 02142, USA; 3 European Molecular Biology Laboratory, EMBL Barcelona 08003, Spain

## Abstract

Throughout the process of vascular growth and remodeling, the extracellular matrix (ECM) concurrently undergoes significant changes due to proteolytic activity—regulated by both endothelial and surrounding stromal cells. The role of matrix metalloproteinases has been well-studied in the context of vascular remodeling, but other proteases, such as cysteine cathepsins, could also facilitate ECM remodeling. To investigate cathepsin-mediated proteolysis in vascular ECM remodeling, and to understand the role of shear flow in this process, *in vitro* microvessels were cultured in previously designed microfluidic chips and assessed by immunostaining, zymography, and western blotting. Primary human vessels (HUVECs and fibroblasts) were conditioned by continuous fluid flow and/or small molecule inhibitors to probe cathepsin expression and activity. Luminal flow (in contrast to static culture) decreases the activity of cathepsins in microvessel systems, despite a total protein increase, due to a concurrent increase in the endogenous inhibitor cystatin C. Observations also demonstrate that cathepsins mostly co-localize with fibroblasts, and that fibrin (the hydrogel substrate) may stabilize cathepsin activity in the system. Inhibitor studies suggest that control over cathepsin-mediated ECM remodeling could contribute to improved maintenance of *in vitro* microvascular networks; however, further investigation is required. Understanding the role of cathepsin activity in *in vitro* microvessels and other engineered tissues will be important for future regenerative medicine applications.

## INTRODUCTION

Vasculogenesis is the formation of *de novo* microvessels usually observed in embryogenesis, tumor growth, and after extensive vascular damage;^1,2^ this differs from angiogenesis, where microvessels are formed from pre-existing vessels, usually after injury.^3^ These growth processes are complex but well-coordinated, involving ECM remodeling, endothelial cell migration, cytokine secretion, lumen formation, and mural cell (i.e., pericyte) recruitment.^1–3^ The complexities involved in vascular growth, when disrupted, can also lead to vascular regression. While many of these growth processes have been characterized using *in vitro* vessel systems, less research has focused on vessel regression. *In vitro* vascular microenvironments are significantly influenced by their cellular constituents, biochemical gradients, and hydrogel substrates within which they are cultured, as well as the mechanical cues imparted on the systems.^4^ The mechanisms driving these changes require clear investigations to aid in the generation and long-term maintenance of vascularized tissue-engineered substrates.

In particular, dynamic changes in the ECM heavily influence both vascular formation and remodeling. For instance, capillary morphogenesis requires proteolytic remodeling of the ECM to allow for endothelial migration and invasion into perivascular tissue, as well as for tubulogenesis (lumen formation) to occur.^3,5,6^ This process is mediated by various cell types secreting angiogenic factors and extracellular proteases, such as matrix metalloproteases (MMPs).^3,5,7^ Basement membrane and other ECM are involved in regulating this process; acting as a reservoir for bound growth factors and proteases, which could indirectly regulate endothelial cell behavior.^3^ Vascular-associated MMP activity has been well-studied and characterized. Following induction by pro-angiogenic factors (such as VEFG and FGF), MMPs are secreted by endothelial cells and degrade the surrounding ECM.^3,7^ Both, *in vitro* and *in vivo* models have demonstrated the importance of MMPs in migration and invasion of endothelial cells.^8,9^ Specifically, MMP-1, -2, and -9, degrade the ECM and endothelial basement membrane,^10^ and can also release ECM-bound angiogenic growth factors.^11^ Tethered MMPs regulate pericellular fibrinolysins; specifically membrane type-1 MMP (MT1-MMP) on the membrane of endothelial cells is required for pericellular fibrinolysis during neovessel formation.^12^ It has also been reported that MT1-MMP dependent proteolysis is important for lumen formation in collagen matrices, a process that is necessary for endothelial cell network formation.^13^ As such, MMPs have been a common focus of study^7,10,11^ and a primary target of inhibition during the development of engineered microvessels, as one strategy to enhance and/or prolong their development.^14,15^ However, other proteases, like cysteine cathepsins, probably contribute to active ECM remodeling in the vascular niche and have been largely ignored.

Cathepsins are amongst the most potent mammalian collagenases and elastases.^16–18^ Cathepsin activity has been implicated in angiogenesis and in the pathogenesis of vascular diseases, including atherosclerosis and the formation of abdominal aortic aneurysms.^19–26^ Studies have shown that cathepsins K and S are involved in neovascularization, with their inhibition impairing normal vessel formation.^20,21^ For instance, stimulation of endothelial cells with inflammatory cytokines and angiogenic factors promotes cathepsin S expression; however when cathepsin S is inhibited, endothelial cell invasion and tube formation is impaired.^19^ Increased expression of cathepsin L has also been linked to cell invasion and neovascularization, and has been identified in invasive and migratory endothelial phenotypes (tip and stalk cells).^27–29^ Thus, cathepsins potentially play a pivotal role in vascular-associated ECM remodeling.

Here, we explore whether cathepsin expression and activity could contribute to remodeling *in vitro* microvascular networks and their pericellular niche. To do so, we employ a previously designed microfluidic model to examine cysteine cathepsins S, K, and L, in a co-cultured vascular system of human umbilical vein endothelial cells (HUVECs) and lung fibroblasts. Cathepsins are interrogated in response to shear flow and small molecule inhibition both on-chip and in 2D cultures, by immunostaining, western blotting, and zymography. Our work demonstrates that shear flow decreases overall cathepsin activity through an upregulation of an endogenous inhibitor, cystatin C. Moreover, fibroblasts contribute significantly to the expression of cathepsins, more so than endothelial cells. Exogenous addition of a cathepsin inhibitor is observed to reduce activity in microvessels, suggesting that cathepsin interventions could potentially be used to prolong *in vitro* microvessel stability. Our results demonstrate the need to further regulate cathepsins, as they contribute to ECM remodeling in microvascular networks.

## RESULTS

### Luminal (shear) flow through microvessels decreases active cathepsins and increases cystatin C

**FIG. 1.**
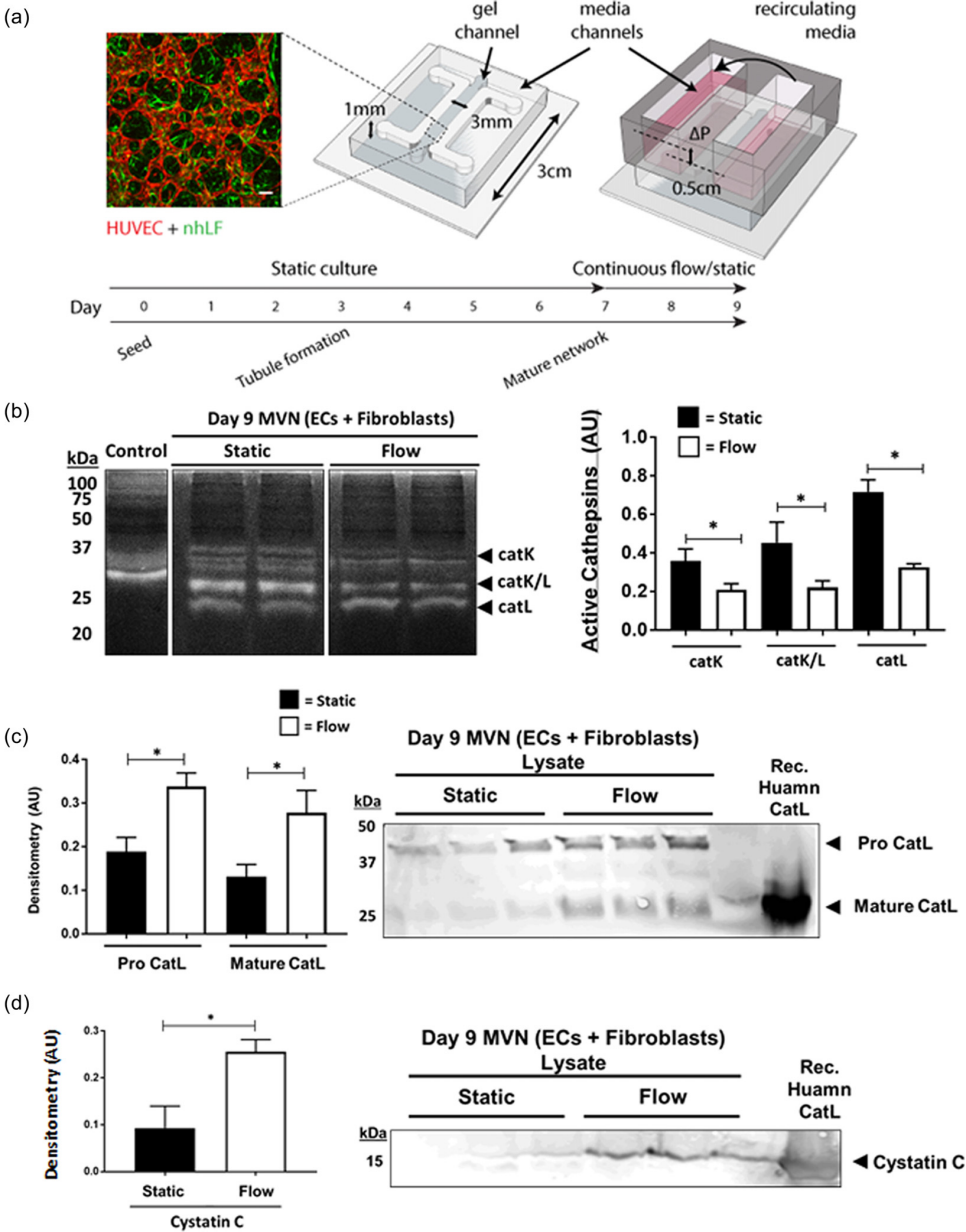
Luminal (shear) flow through microvessels decreases active cathepsins and increases cystatin C. (a) ECs and fibroblasts were cultured at a 5:1 ratio in a 3 mg/ml fibrin gel within a macro-scale (∼100 μl gel volume) Polydimethylsiloxane (PDMS) platform. Microvessels (MVNs) formed via a vasculogenesis-like process over ∼7 days. Scalebar shown is 100 *μ*m. Some devices were exposed to continuous flow, inducing a low shear stress (∼0.5 Pa) for 48 h. Flow was maintained by a pressure drop generated across the gel using an in-house pump. Two experiments each with three biological replicates for static conditions and four biological replicates for flow conditions were completed. Gels containing MVNs were extracted from devices (static and shear) on day 9. (b) Zymography was run to assess the amount of active cathepsins, as indicated by white clear bands and western blots were done to assess for the presence of cathepsins and cystatin C. Immunoblots and zymograms were quantified via densitometry; error bars in quantified densitometry represent SEM. (b). Multiple active cathepsin bands were observed in the zymogram. There were significantly less active cathepsins in MVNs formed under flowshear stress compared to static devices (p < 0.05). Western blots were done to assess for (c) presence of cathepsins and (d) cystatin C. There was significantly more pro (p = 0.0049) and mature cathepsin L (p = 0.0332) in networks formed under flow compared to static conditions. Cystatin C was significantly increased in MVNs formed under flow (p = 0.0219).

By generating *in vitro* 3D human vessels on-chip, we are able to control the environment to examine cathepsin activity in response to applied fluid shear stresses. Considering the significant influence that fluid flow (interstitial and shear) has on vasculature,^35,40,41^ and how mechanical cues are tied to remodeling of the vascular niche,^42,43^ it is important to investigate how these mechanisms alter cathepsin activity. It has been previously reported that cathepsins are mechanosensitive.^20^ For instance, cathepsin L activity is inhibited when endothelial cells are exposed to laminar shear stress.^44^ We hypothesized that luminal flow applied to cultured perfusable vessels would reduce active cathepsins in microvascular networks. To test this hypothesis, microvascular networks were developed in a large-scale version of a single-gel channel PDMS device (similar to our previous designs) using a co-culture of human umbilical vein endothelial cells (HUVECs) and normal human lung fibroblasts in a fibrin gel, as previously described.^32,33^ Lung fibroblast co-cultures were chosen for their ability to result in relatively stable vessel formation with HUVECs (∼1–2 weeks in culture), as opposed to other cells, including pericytes. Following microvessel formation, pressure driven recirculating fluid flow was applied from day 7 to 9 (48 h) in 50% of the devices, while the other 50% were cultured under static conditions (controls). A custom pump was used to maintain a pressure drop across the gel channel containing the microvessels, imposing a mean shear stress of ∼0.5 Pa or 5 dynes/cm^2^ [Fig. 1(a)].^31^ Following flow-conditioning, the microvessel networks were collected (extracted from the chip) and homogenized, then samples were prepared for cathepsin zymography. Multiple active cathepsins bands were detected (37 kDa, ∼27 kDa, and ∼23 kDa), which were determined to be cathepsins (cat)K or catL, based on known electrophoretic distances.^35^ Flow conditioning significantly reduced the amount of active catK (−42% ± 2.3%), catK/L (−52% ± 4.5%), and catL (−55% ± 4.6%) [Fig. 1(b)].

Zymograms were repeated, in the presence of either a catK inhibitor or a catL inhibitor (supplementary material Fig. S1) to confirm the presence of bands seen in Fig. 1(b). Incubation with the catK inhibitor blocked the appearance of the 37 kDa, 27 kDa, and 23 kDa active bands. In the presence of the catL inhibitor, the 37 and 23 kDa active bands no longer appeared; however, microvascular networks subjected to flow retained some active cathepsin at 27 kDa, compared to static conditions (p = 0.0134). Thus, the 37 kDa, 27 kDa, and 23 kDa bands are expected to be catK, and the 37 and 23 kDa bands are catL.

Although zymography is used to detect active cathepsins, immunoblots are required to detect the total protein content. It is possible that the pro and mature forms of cathepsin could be present, but not in an active conformation yielding a zymography signal; therefore, the total protein was determined by western blot. Flow conditioning of vessels resulted in a 44% ± 0.14% increase in pro-catL levels and 53% ± 2.57% increase in levels of mature catL compared to static controls [Fig. 1(c)]. This result contrasts the amount of active cathepsin detected by zymography [Fig. 1(b)]. To resolve this discrepancy, immunoblots were performed for cystatin C, the tight binding endogenous inhibitor of cysteine cathepsins, which is expressed by most nucleated cells, and previously shown to be upregulated by endothelial cells under flow conditions.^45^ Consistent with these results, microvascular networks subjected to flow demonstrated a 64% ± 13% increase in cystatin C compared to those cultured under static conditions [p = 0.0219, Fig. 1(d)]. This suggests that flow conditioning upregulates cystatin C which can bind to cathepsins and reduce the amount of free, active cathepsins, thus aligning with results Fig. 1(b). We acknowledge in lane 3 of Fig. 1(c) that the pro-catL band appears darker in the immunoblot compared to lanes 1 and 2. This observation is likely due to biological variability, still, there is no corresponding increase in mature catL, or the active form capable of proteolysis,^46,47^ compared to flow conditions.

### Fibroblasts remodel the microvascular niche and co-localize with cathepsins K, L, and S

**FIG. 2.**
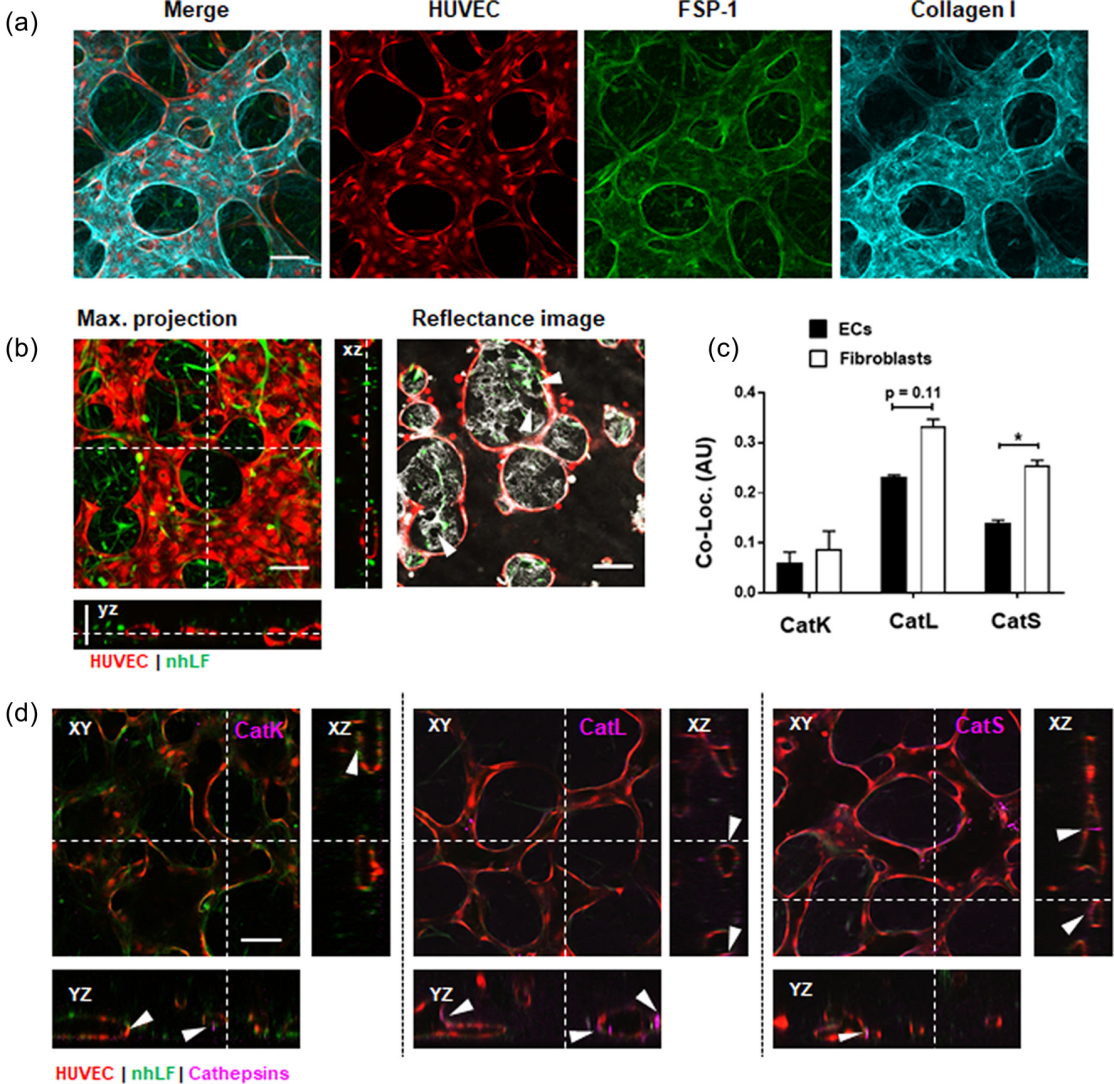
Cathepsins localize to the lumen and the surrounding vessel stroma. (a) Confocal images of day 7 (show RFP)-labeled-expressing HUVECs, with immunolabeled FSP-1 to identify normal human lung fibroblasts (nhLFs), and immunolabeled collagen I. (b) Left image is a live maximum projection confocal image showing open vascular lumens in corresponding orthogonal projections. Right image is a single plane of the maximum projection from the left, shown to demonstrate matrix remodeling (white regions are corresponding reflectance of the matrix and arrows demonstrate degradation). (c) A co-localization plugin (NIH ImageJ) was used to identify the overlapping intensity between endothelial cells (ECs) or fibroblasts and catK, catL, or catS from stained images, as shown in (d). Cathepsins co-localize significantly with fibroblasts compared to ECs. Significance is indicated by *p < 0.05 using a *t* test compared between ECs and fibroblasts for each cathepsin. (d) Immunostains of microvessels identified that catK, catL, and catS were localized in the endothelium on the apical and basal sides (white arrows), and in the stroma where fibroblasts are located (shown by white arrows). All scale bars are 100 *μ*m.

We next used immunofluorescence staining to visually confirm the co-localization of cathepsins in the microvessels and surrounding stroma (Fig. 2 and supplementary material Fig. S2). Day 7 microvascular networks containing RFP-labeled HUVECs were first fixed and stained with antibodies to demonstrate the localization of fibroblasts and the deposition of ECM protein. Fibroblast specific protein (FSP-1) was used to label fibroblasts, which both wraparound vessels, as well as distribute throughout the stroma. Collagen I is shown to be highly localized with vessels and fibroblasts [Fig. 2(a)]. In addition to collagen deposition, fibroblasts degrade the surrounding fibrin hydrogel [indicated in Fig. 2(b) by white arrows]. The activity of fibroblast remodeling corresponds with their increased co-localization that we see with cathepsins [Fig. 2(c)]. A co-localization plugin (NIH ImageJ) was used to identify the overlap in intensity between endothelial or fibroblast cells and catK, catL, or catS. Briefly, mean co-localization R values (acquired from z projections of n = 3 images each) showed that cathepsins L and S localize predominantly with fibroblasts, in comparison to endothelial cells [Fig. 2(c)]. We identified that catK, catL, and catS were localized with the endothelium as well, particularly where fibroblasts were situated [Fig. 2(d)].

### Pan-protease inhibitors abruptly reduce cathepsins in vessels on-chip

**FIG. 3.**
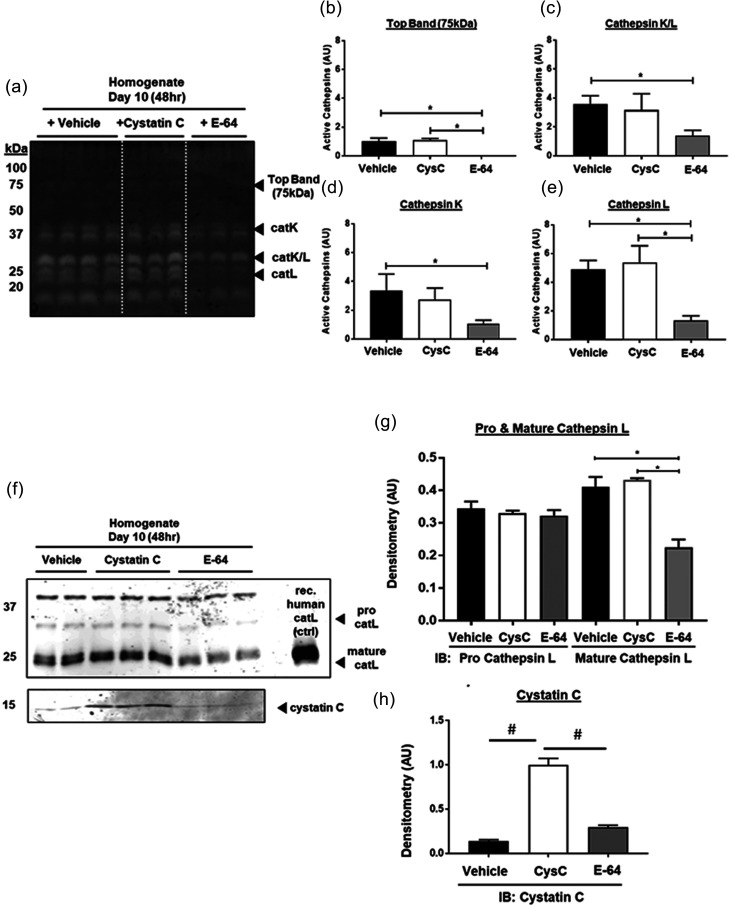
Cathepsin inhibitor, E-64, reduces active cathepsins in microvessels. MVNs were treated with cathepsin inhibitors, cystatin C or E-64, from day 8 to day 10 under static conditions. Lysate, containing MVNs was collected. One experiment with four biological replicates for control conditions and three biological replicates for E-64 or cystatin C inhibitor conditions was completed. (a) Cathepsin zymography was used to measure the amount of active cathepsins in the microvessel. Error bars in quantified densitometry represent SEM. (a) E-64 treated MVNs, compared to control MVNs, had (b) significantly reduced active top band (75 kDa, p = 0.0004), (c) catK (37 kDa, p = 0.0271), (d) catK/L (27 kDacatL (25 kDa, p = 0.0155), and (e) catL (23 kDa, p = 0.0012) in lysate, which contains microvessels, cells, and matrix components. There was no significant difference between cystatin C-treated and control networks. (f) Western blots of the lysate showed that there was no significant difference in pro-catL between the three treatment groups. (g) E-64 treated MVNs have significantly less mature catL compared to control (p = 0.0026) and cystatin C (p = 0.0021) treated MVNs. (h) At day 10, there was significantly more cystatin C in MVNs treated with cystatin C compared to control (#p < 0.0001) or E-64 (#p < 0.0001).

To test if exogenously applied inhibitors could reduce active cathepsins in microvascular networks, we exogenously employed two cysteine cathepsin inhibitors: cystatin C, typically an endogenous 13 000 Da protein that is a broad cathepsin inhibitor, and E-64, a 357 Da broad-spectrum inhibitor of cysteine cathepsin family members.^48^ On day 8, after microvascular networks were formed, either cystatin C (1 *μ*M), E-64 (10 *μ*M), or control (equal volume vehicle) was added to microvascular network cultures that were cultured under static conditions for 48 h to allow time for the inhibitors to equilibrate and bind to any cathepsins already present, plus any newly synthesized cathepsins. Homogenates (vessel lysates) and supernatants were collected to quantify the amount of cell-associated and secreted active cathepsins, respectively, in the presence of these inhibitors [Fig. 3(a) and supplementary material Fig. S3]. Pan cathepsin inhibition with E-64 significantly reduced the top active band (75 kDa, p = 0.0004), active catK (37 kDa, −68% ± 9.7%, p = 0.0271), catK/L (27 kDa, −61% ± 8.7%, p = 0.0155), and catL (23 kDa, −73% ± 10.4%, p = 0.0012) compared to microvascular networks with no inhibitor added [Figs. 3(b)–3(e)]. Cystatin C treatment did not significantly reduce the amount of active cathepsins detected, and is suspected to be due to the low concentration (which has shown an effect previously in 2D cultures^34^).

Immunoblots of these samples quantified total protein of catK, catL, and catS, following treatment with cathepsin inhibitors [Fig. 3(f)]. CatK and catS were not detectable by immunoblot. From lysates, E-64 significantly reduced the amount of mature catL in microvessels compared to cystatin C (p = 0.0021) or controls (p = 0.0026). There was no significant difference in pro-catL between the three groups [Fig. 3(g)]. There was significantly more cystatin C in vessels treated with the exogenous cystatin C, compared to controls (86% ± 12.3%, p < 0.0001) and E-64 treatment (71% ± 10.1%, p < 0.0001) [Fig. 3(h)].

**FIG. 4.**
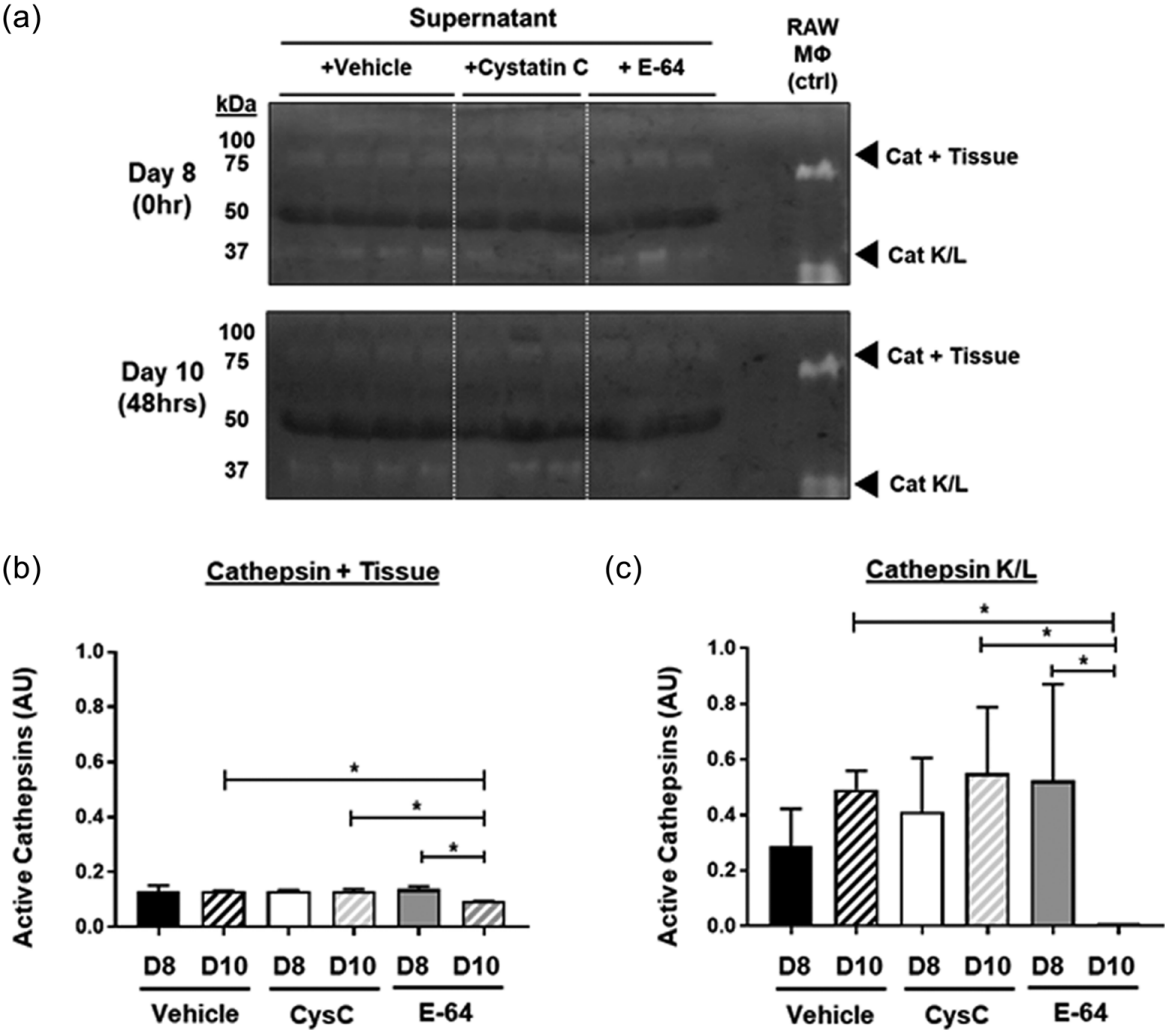
E-64 treatment reduces cathepsin activity overall in supernatants through MVNs. MVNs were treated with cathepsin inhibitors, cystatin C or E-64, from day 8 to day 10 under static conditions. Supernatant, containing media, was collected from devices. Cathepsin zymography was used to measure the amount of active cathepsins in the microvessel. (a) In the supernatant, there was a significant decrease in the amount of active cathepsins between day 8 and 10 in the microvessels treated with E-64 (p = 0.0448). (b) Zymography also detected cathepsin bound to tissue (75 kDa). (c) At day 10, there was significantly less active catK/catL in E-64 treated samples compared to control (p = 0.0442) and cystatin C (p = 0.0326).

Similarly, in the supernatant, there was nearly a complete reduction in the amount of active cathepsins between 0 and 48 h (Fig. 4) in microvascular networks treated with E-64 (p = 0.0448). After 48 h of inhibition, there was significantly less active catK/L in E-64 samples compared to the cystatin C group (p = 0.0326) and controls (p = 0.0442) (Fig. 4). Zymography also detected active cathepsin bands at 75 kDa, which was reduced by 32% ± 3.9% after addition of E-64; however, there was little difference in the amount of active cathepsins between control networks and those treated with exogenous cystatin C in the supernatant. We have previously identified 75 kDa bands as active cathepsins bound to the ECM and/or tissue fragments,^49^ and so it is also possible that cathepsin could be bound to fibrin gels.^50^

Likewise, the supernatant of microvascular networks with cystatin C for 48 h (day 10) had a threefold higher cystatin C level, compared to control (p < 0.0001) and E-64 treated vessels (p < 0.0001) (supplementary material Fig. S3), clearly indicating that broad uptake of exogenous cystatin C had not ensued. Despite there being significantly more cystatin C present in the supernatant after exogenous supplementation, there was no decrease in the amount of active cathepsins in the homogenate suggesting there was not endothelial transcytosis of cystatin C from the supernatant to the basal side of microvessels.

### Fibroblasts are a main source of cathepsins—the activity of which is sustained by fibrin

**FIG. 5.**
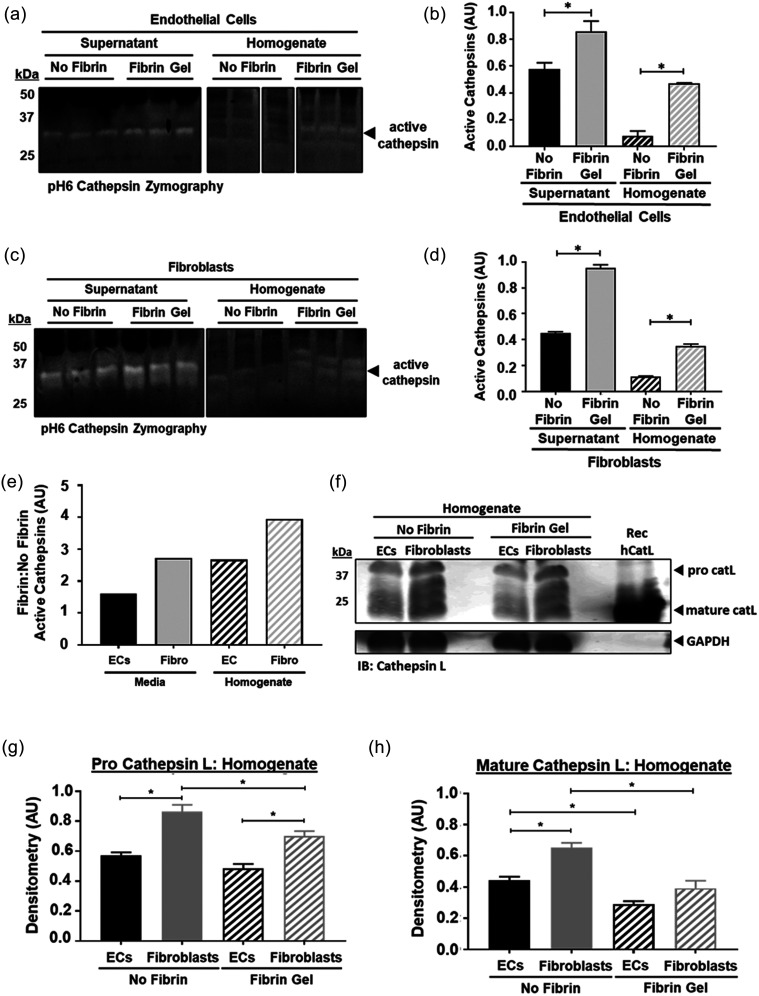
Fibrin and fibroblasts sustain active cathepsins in microvascular networks. (a) and (b) Endothelial cells (ECs) and (c) and (d) fibroblasts were separately cultured on fibrin gels or tissue culture plates (TCP); homogenate, containing cells and fibrin gel, and media, containing secreted proteins, were collected and prepared in non-reducing loading dye. One experiment with three biological replicates for HUVECs and three biological replicates for fibroblasts was completed). Multiplex cathepsin zymography was run to assess the amount of active cathepsins. Immunoblot and zymogram were quantified via densitometry; error bars in quantified densitometry represent SEM. When ECs or fibroblasts are cultured on fibrin gel, there are increased amounts of active cathepsins in media (p = 0.0023, p < 0.0001) and homogenate (p < 0.0001, p < 0.0001) for cells cultured on fibrin gel. (e)–(g) Western blots were also used to quantify cathepsins in the supernatant and homogenate. (e) Based on the ratio of active cathepsins on fibrin compared to no fibrin, fibroblasts have more active cathepsins compared to ECs. (f)–(h**)** In homogenate, fibroblasts have each cell type, and consistently fibroblasts significantly have more cathepsins than ECs.

Endothelial cells and fibroblasts are initially co-cultured in fibrin gels, and over time as endothelial cells coalesce they begin to form networks and integrate with fibroblasts in the surrounding stroma. Doing so requires remodeling and deposition of ECM proteins, and both cell types deposit matrix such as collagens over time, as we earlier demonstrated [Fig. 2(a)]. Studies have shown that substrates such as collagen and elastin can sustain and extend cathepsin activity through their adsorption following cellular release.^51,52^ Our previous work also demonstrates fibrin as a substrate that can sustain active cathepsins for 24 h.^50^ We hypothesized that the ECM of our 3D microvascular networks could serve as a bioactive reservoir to sustain cathepsin activity. Therefore, endothelial and fibroblast cells were separately cultured on fibrin gels for 24 h (well-plates). Both homogenate (containing both cell lysate and fibrin) and supernatant were collected. Multiplex cathepsin zymography was used to identify active cathepsins in the supernatant and homogenate of the individually cultured cell types. Endothelial and fibroblast cells cultured on fibrin expressed significantly more active cathepsins compared to those cultured on standard un-coated plates [Figs. 5(a)–5(d)]. By calculating the ratio [Fig. 5(e)] of active cathepsins (fibrin-coating: no fibrin-coating) for both cell types, it is clear that fibroblasts (4:1 in homogenate, 3:1 in media) demonstrate increased cathepsin activity compared to endothelial cells (3:1 in homogenate, 2:1 in media ratio).

Endothelial cells and fibroblasts might increase cathepsin production in the presence of fibrin, which could be facilitated by either gene-expression changes induced by integrin binding,^53^ or by promoting localization of cathepsins to the cell surface.^54^ HUVECs and fibroblasts were separately cultured on fibrin-coated or non-coated tissue culture polystyrene for 24 h. CatK and catS were not detectable by immunoblot, despite observations in immunostained samples (supplementary material Fig. S4); however, catL was identified. Fibroblasts produced more pro-catL and mature catL compared to endothelial cells [Figs. 5(f)–5(h)], which supports our findings that cathepsins mostly co-localize with fibroblasts [as seen in Fig. 2(c)]. Interestingly, the homogenate, which contains cell lysate, fibrin gel, and any proteins associated/bound to them, contained less pro-catL and mature catL in fibroblasts cultured on fibrin gels compared to no-fibrin [Figs. 5(F)–5(h)]. The opposite was found in the supernatant, where fibroblasts (and separately endothelial cells) cultured on fibrin gel produced more unbound pro-catL and mature catL (supplementary material Fig. S5).

## DISCUSSION

Proteolytic activity is required for matrix and vascular remodeling to occur—and we proposed and demonstrated herein that cathepsins actively contribute to this process. To date, researchers have focused on proteolysis due to MMPs as proteases;^14,15,55–57^ however, another class of proteases, cysteine cathepsins, need to be considered and have been also implicated in vascular remodeling.^19–21,25^ MMPs and cathepsins are both secreted by endothelial and fibroblast cells;^29,58–60^ the two cell types often co-cultured to generate vessels on-chip. Both protease types interact via coordinated and cascading events, with interactions occurring between familial groups.^61^ Understanding proteolytic interactions could help us uncover the role of cathepsins in vascular remodeling, and to identify approaches to improve tissue-engineered vessels. Here, we aimed to reveal cathepsin expression and activity in microvessels, and to identify how they are regulated.

First, we generated 3D microvessels using our *in vitro* microfluidic systems to investigate the presence of cathepsins and their activity. Importantly, we show that cathepsins are expressed in vessels and that their activity levels are highly dependent on the culture environment (cell composition, mechanical cues, and substrate). Microvessels generated in a fibrin matrix express significant levels of active catK and catL, which we found were predominantly produced by fibroblasts. CatK, catL, and catS localize with vessels, as well as fibroblasts, which associate with them. Fibroblasts produce considerably more cathepsins compared to endothelial cells (as determined by complementary 2D assays), validating our observations of increased co-localization with fibroblasts in 3D vascular networks. Moreover, cathepsin expression was shown to be directly influenced by the substrate—with fibrin gel promoting cathepsin levels and activity. The extracellular component of the vascular stroma was shown to act as a bioactive reservoir, which can sustain proteases—making them available for matrix degradation later on. This delayed, substrate-mediated, effect could contribute to regression of the vasculature if uncontrolled matrix degradation takes place over time. Our work demonstrates the need to consider novel cathepsin inhibition strategies to control proteolysis for fibrin-based engineered vessels.

Fluid shear stress is known to regulate MMP activity, and has also been shown to regulate cathepsin activity *in vitro*. In particular, laminar shear stress has been previously shown to inhibit catL activity in mouse aortic endothelial cells.^44^ The flow profile (laminar vs turbulent) and rate of flow can also affect cathepsin activity. For instance, endothelial monolayers exposed to pro-atherogenic oscillatory flows at 5 dynes/cm^2^ resulted in higher catK expression, compared to athero-protective unidirectional shear stress (15 dynes/cm^2^).^26^ Various microfluidic strategies have been used to create perfusable microvasculature on-chip, similar to ours, due to the afforded control that these systems offer.^4^ Our system was connected to a media reservoir and a pump designed to impart continuous flow across our hydrogel and through our pre-formed perfusable vessels. By generating a pressure gradient, shear flow (∼5 dynes/cm^2^) was maintained across the vessel system for 2 days. Overall, flow induced a decrease in cathepsin activity in our system, which at first seemed counterintuitive. However, flow increased total cathepsins present in the system, and also increased the total amount of cystatin C—thereby resulting in reduced active cathepsins in microvessels. Previous reports also suggest that flow alters both cathepsin expression and cystatin C in endothelial cells.^45^ Cathepsin isoforms (catK and catL) were also shown to be regulated differently by flow. We aim to assess varied flow conditions in our future work, as it is clear that shear flow regulates activity of cathepsins in vascular networks.

To test whether we could control cathepsin activity, we exogenously added the broad-spectrum protease inhibitors, cystatin C or E-64, to our 3D microvessels. Cystatin C is the endogenous enzyme that reversibly binds to active sites of cathepsins to prevent substrate degradation.^62^ E-64 is small molecule inhibitor that irreversibly binds to the cathepsin active site.^48^ Addition of E-64 to microvascular networks significantly reduced active cathepsins, in contrast to exogenously applied cystatin C, which had no effect. Cystatin C is an extracellular cathepsin inhibitor; and its use resulted in cathepsin presence restricted mostly in the supernatant, suggesting that the vessels may have presented a strong barrier against transendothelial transport. It is probable that the molecular size of the inhibitors (13 kDa for cystatin C, vs 0.35 kDa for E-64) causes differences in cellular uptake through pinocytosis and transport, as we have previously shown with dextrans of varied size.^32^ Our lab has previously observed upregulated cathepsin activity (catL) in other multi-cellular engineered living systems (bio-bots) employing fibrin as a key matrix component, and demonstrated that treatment with E-64 could extend their lifetime in culture.^63^ Thus, cathepsin inhibition may improve longevity of other tissue-engineered constructs, in addition to *in vitro* vessels—an approach we are investigating in on-going work.

In addition to cytokines and shear stress, it is known that cell–cell interactions with the matrix can regulate protease expression and activity, as we have previously shown.^26,44^ A key observation herein is that fibroblasts produce more cathepsins than endothelial cells in our microvessel systems, and their activity is mediated by the presence of fibrin substrates (Fig. 5). Interestingly, stromal cells (like fibroblasts) are essential for promoting and maintaining microvessels *in vitro*, and help to improve their patency.^42,43,64–66^ Eliminating fibroblasts to reduce the number of active cathepsins may not be conducive for maintaining long term vessel viability; however, as we demonstrate, the addition of continuous flow and protease inhibitors can decrease active cathepsins to prevent proteolysis. This could make fibroblasts (or other stromal cells) an ideal cell target for creating inducible cell lines to control endogenous cathepsin production. This could be done by creating a “switch” to turn on cathepsins to facilitate remodeling, with the ability to switch off cathepsin production when activity starts becoming destructive. Also, one could consider using nanoparticle microcarriers for timed delivery of E-64, seeing that this was the optimal inhibitor for cathepsins in our microvessels.

It appears that vascular regression is in-part due to unbalanced proteolytic activity. Adding broad spectrum MMP inhibitors, Zhu *et al.* demonstrated a time-dependency on vessel formation in an aorta-angiogenesis (rat) assay. When added at the beginning of the angiogenic growth phase, microvessel formation was completely blocked; however, addition afterward prevented vessel regression.^67^ Since we did not see prolonged vessel growth following the addition of pan-proteolysis inhibitors in our system (data not shown), it is possible that we may have blocked proteolytic activity too-late. In another example, a broad spectrum MMP inhibitor and/or serine inhibitor (inhibits plasmin, the endogenous fibrin-degrading enzyme) was used in an *in vitro* bead-angiogenic assay. Interestingly, when used alone, neither inhibitor had a significant effect on vessel sprouting; however, the combination of both inhibitors completely blocked angiogenic sprout formation in fibrin gels.^55^ We have also previously observed active catL in bio-bots (tissue-engineered systems in fibrin gels), despite their treatment with a serine protease inhibitor aminocaproic acid.^49^ These results suggest that an additional protease family, such as cysteine cathepsins, is an active contributor in fibrin degradation. It will be important to perform cathepsin loss and gain of function studies in endothelial and stromal cells in future work to tie cathepsin activity directly to each cell type in the context of vessel remodeling. Understanding cathepsin activity in the larger proteolytic network is important for developing strategies to control remodeling in tissue engineered systems, such as the *in vitro* vascular niche.

## CONCLUSIONS

Overall, we demonstrate that cathepsin activity is highly regulated within tissue-engineered microvessels. Our work is the first, to the best of our knowledge, to interrogate cathepsin expression and activity in 3D vessels in response to both biochemical and biomechanical cues. Shear flow was confirmed to be a significant moderator of cathepsin-mediated signaling within the vascular bed. Cathepsin expression was increased by shear-flow, as is the expression of its endogenous inhibitor, cysteine C. Furthermore, cathepsins may bind to the substrate (fibrin here) and prolong their activity. We also demonstrate that small molecular inhibitors are capable of blocking cathepsin activity in our system. We postulate that blocking cathepsin activity prior to microvessel regression may prolong their viability in culture. Taken together, our work highlights the importance of cathepsin activity in ECM remodeling of vascular *in vitro* systems. Future work will be necessary to confirm the potential for cathepsin blocking strategies to effectively stabilize the engineered vascular niche.

## METHODS

### Cell culture and generation of 3D microvascular networks

Human umbilical vein endothelial cells (HUVECs) were transduced by standard lentivirus procedures (as described previously^18,30^) to label the cytoplasm with red or blue fluorescent protein (RFP or BFP). Non-fluorescent normal human lung fibroblasts were used in all experiments, except when cytoplasmic GFP expressing-fibroblasts were used for visualization purposes. Both primary human cell types were purchased from Lonza and were cultured until confluency in flasks coated with rat tail collagen I (50 mg/ml, Corning). HUVECs were cultured in VascuLife media (LL-0003) and fibroblasts were cultured in FibroLife (LL-0011) media (Lifeline Cell Technology) and completely refreshed every two days.

Once confluent, cells were dissociated using TrypLE (ThermoFisher) and re-suspended in thrombin, separately, to concentrations of 24 million endothelial cells/ml and 4.8 million fibroblasts/ml. Cell suspensions were mixed 1:1 by volume and then mixed with fibrinogen solution. This results in a final concentration of 6 million endothelial cells/ml and 1.2 million fibroblasts/ml, in a 5:1 HUVEC:fibroblast ratio within fibrin (3 mg/ml). The cell-gel solution was injected within a macro-scale (∼100 *μ*l total gel volume) PDMS (10:1 ratio, Ellsworth adhesives) single-gel channel device [see Fig. 1(a)]. Microvascular networks formed via a vasculogenesis-like process over ∼7 days, following which half of the devices were exposed to continuous flow, inducing a low shear stress (∼0.5 Pa = 5 dynes/cm^2^—mean value across vessels, as determined previously^31^) for 48 h. Flow was maintained by a pressure head (∼0.5 cm H_2_O) generated across the gel using a fluid chamber fit to the PDMS device, and media was re-circulated to maintain this pressure head by an in-house built pump. Sample collection for immunoblot and zymography was performed by extracting the hydrogel component of the PDMS devices containing microvascular networks (for both static and flow conditions) on day 9. These samples were flash-frozen in the vapor phase of liquid nitrogen, and then stored at −80 °C until further sample preparation as indicated below. Two experiments with three biological replicates for static conditions and four biological replicates for flow conditions were completed. Each set of biological replicates was run on its own immunoblot or zymogram.

### 2D cell culture on fibrin substrates

Fibrin gel (3 mg/ml) was prepared by mixing at a 1:1 ratio 6 mg/ml fibrinogen (bovine plasma, Sigma) with 4 U thrombin (bovine plasma, Sigma). A total volume of 100 *μ*l was added to the center of each well of a 12-well plate and rotated for complete coating. Fibrin gelation occurred over several minutes, prior to the addition of complete media and incubation at 37 °C overnight in a standard incubator. The following day, endothelial and fibroblast cells were seeded onto either non-coated or fibrin-coated wells, at a concentration of 5 × 10^4^ cells/well. HUVECs and fibroblasts were then cultured in VascuLife or FibroLife, respectively. Following 48 h, media were collected into a separate tube. The remaining cells and fibrin gel (homogenate) were collected and homogenized in lysis buffer. One experiment with six biological replicates for HUVECs and six biological replicates for fibroblasts (three replicates used for electrophoresis and three replicates used for immunostaining for both cell types) was performed.

### Immunofluorescence staining and confocal microscopy

Microvascular networks in microfluidic devices were fixed at day 7 using 4% paraformaldehyde [PFA, 8% Electron Microscopy Science, diluted with Dulbecco's phosphate buffered saline (DPBS), VWR] for 30 min. Well-plates were fixed at day 2. Following several washes with DPBS, samples were subsequently permeabilized with Triton-x 100 (0.1% v/v in DPBS) for 10 min and incubation in blocking buffer [5% w/v BSA (Sigma), 3% v/v goat (or horse) serum in DPBS] for several hours. Primary anti-human antibodies were used at 2 *μ*g/ml in wash buffer (0.5% w/v BSA in DPBS) as follows: rabbit polyclonal anti-cathepsin K (Proteintech), goat polyclonal anti-cathepsin L (R&D systems), goat polyclonal anti-cathepsin S (R&D systems), anti-collagen 1 (Abcam, ab34710), and monoclonal mouse anti-FSP1 (Novus Biologicals). Appropriate secondary antibodies (ThermoFisher) were used at dilutions of 1:200. DAPI (Invitrogen) was used at 1:1000 for 10 min. Samples were imaged on a confocal microscope (Olympus IX81) using Fluoview v4.1. Z-stack images were captured using 5 *μ*m slices (∼20–30 total XY-images) and presented as maximum z-projections, unless otherwise specified. Co-localization of antibodies was performed using the NIH ImageJ analysis tool *Colocalization thresholds*.

### Cathepsin inhibitor experiments

Microvascular networks were grown in a scaled-down version (same as prior use^32,33^) of the microfluidic chip (∼35 *μ*l gel volume). These microvascular networks were cultured until day 7, prior to the addition of 10 *μ*M E-64 (Calbiochem/Millipore Sigma) or 1 *μ*M cystatin C (Molecular Innovations),^34^ or a control volume of diluent in the media. These samples were cultured until day 10 and flash-frozen in the vapor phase of liquid nitrogen, and then stored at −80 °C until further sample preparation as indicated. One experiment with four biological replicates for control conditions and three biological replicates for E-64 or cystatin C inhibitor experiments was performed.

### Immunoblotting

Samples were prepared with reduced loading dye (5× − 0.05% bromophenol blue, 10% SDS, 1.5M Tris, 50% glycerol, 25% betamercaptoethanol) and run on 12.5% SDS-PAGE gels. Proteins were transferred onto a nitrocellulose membrane, and then immunoblotted using the following antibodies: rabbit polyclonal anti-cathepsin K (Proteintech), goat polyclonal anti-cathepsin L (R&D Systems), goat polyclonal anti-cathepsin S (R&D Systems), rabbit polyclonal anti-cystatin C (EMD Millipore), or goat polyclonal anti-GAPDH (R&D Systems). Immunoblots were imaged using Odyssey CLx, LI-COR. Densitometry was performed with ImageJ (NIH).

### Multiplex cathepsin zymography

Multiplex cathepsin zymography was used to assess active cathepsins, as previously described.^35^ Samples were prepared using non-reducing loading buffer (5 × −0.05% bromophenol blue, 10% sodium dodecyl sulfate (SDS), 1.5M Tris, and 50% glycerol). A 12.5% SDS-PAGE gel was used and embedded with a 5 mg/ml gelatin substrate. Gels were run at 200 V at 4 °C. Proteases were renatured in 65 mM Tris buffer pH 7.4 with 20% glycerol for 3 washes, 10 min each, then incubated in pH6 (phosphate buffer, 1 mM EDTA with 2 mM DTT, freshly added) activity assay buffer overnight. Next, gels were stained with Coomassie Blue (4.5% Coomassie blue; Sigma-Aldrich, 10% acetic acid, and 10% isopropanol), then destained (10% acetic acid and 10% isopropanol). In these experiments, the same amount (100 ng) of RAW264.7 macrophage lysate from the same aliquot was loaded as a control for zymograms. Densitometry was normalized to these controls for each gel to make comparisons among replicates run on separate gels. For inhibitor assays, 1 *μ*M of cathepsin K inhibitor II (1-(N-benzyloxycarbonyl-leucyl)-5-(N-Boc-phenylalanyl- leucyl) carbohydrazide [Z-L-NHNHCONHNH-LF-Boc], Calbiochem/Millipore Sigma) or 1 *μ*M of cathepsin L inhibitor (Z-FY-CHO, Calbiochem/Millipore Sigma) was added to the pH6 assay buffer prior to overnight incubation. Cathepsin K and cathepsin L inhibitor concentrations were selected based on previously published work.^36,37^

### Statistical analysis

Biological replicates were run in separate gels, and in order to compare data across gels the same amount of RAW264.7 macrophage lysate or recombinant enzyme was used as a normalizing signal among different gels, as we have done in earlier analyses.^38,39^ Statistical significance was determined using a one-way ANOVA with Tukey post-hoc analysis for multiple comparisons (unless noted otherwise) using GraphPad. Alternatively, significance was determined using a *t* test when comparing between endothelial cells and fibroblasts for each cathepsin (since primary and secondary antibodies differed between samples); this is indicated for those experiments. All experiments shown have at least three biological replicates and a p value < 0.05 was considered statistically significant. Error bars in densitometry graphs represent standard error of the mean (SEM).

### Ethics approval

Ethics approval is not required.

## SUPPLEMENTARY MATERIAL

See the supplementary material for additional data for inhibitor experiments and 2D HUVECs and fibroblasts experiments referenced in the article.

## AUTHORS' CONTRIBUTIONS

S.A.D. and K.H. contributed equally to this work.

## Data Availability

The data that support the findings of this study are available within the article and its supplementary material, but any other information requested may be available from the corresponding author upon reasonable request.
